# Effects of sandblasting and acid etching on the surface properties of additively manufactured and machined titanium and their consequences for osteoblast adhesion under different storage conditions

**DOI:** 10.3389/fbioe.2025.1640122

**Published:** 2025-08-06

**Authors:** Osman Akbas, Amit Gaikwad, Leif Reck, Nina Ehlert, Anne Jahn, Jörg Hermsdorf, Andreas Winkel, Meike Stiesch, Andreas Greuling

**Affiliations:** ^1^ Department of Prosthetic Dentistry and Biomedical Materials Science, Hannover Medical School, Hannover, Germany; ^2^ Lower Saxony Centre for Biomedical Engineering, Implant Research and Development, Hannover Medical School, Hannover, Germany; ^3^ Institut für Anorganische Chemie, Leibniz University Hannover, Hannover, Germany; ^4^ Laser Zentrum Hannover e.V., Hannover, Germany

**Keywords:** additive manufacturing, dental implants, sandblasting and acid etching, SLA, SAE, surface wettability, contact angle, cytocompatibility

## Abstract

**Introduction:**

Additive manufacturing (AM) enables the production of complex, patient-specific titanium implants. However, the as-built surfaces of AM parts often require postprocessing to enhance surface properties for optimal osseointegration.

**Methods:**

This study investigates the effects of varying sandblasting pressures (2 bar vs. 6 bar) and subsequent acid etching (SAE) on the surface properties of additively manufactured and machined titanium (Ti-6Al-4V and commercially pure titanium (cp-Ti), respectively). While changes in surface roughness and morphology were assessed at different process stages using optical profilometry and scanning electron microscopy, the analyses of surface wettability (contact angle measurement) were focused on effects after SAE and during different storage conditions (ambient air vs. NaCl). The resulting differences in material properties were then evaluated for their biological impact on osteoblast compatibility. For this purpose, the parameters cell adhesion, morphology, and membrane integrity were investigated using confocal laser microscopy and LDH assay.

**Results:**

Initial high roughness of AM titanium surfaces was decreased by sandblasting, while initial smooth machined surfaces (MM) increased in roughness. Acid etching introduced characteristic irregular patterns on the surface with only marginal consequences for the resulting overall roughness. While all surfaces demonstrated high hydrophilicity directly after etching, storage under ambient air increased hydrophobicity over time, while NaCl storage preserved hydrophilicity and improved biocompatibility marginally. Osteoblast adhesion and morphology were optimal only under no storage condition, with uncompromised membrane integrity.

**Discussion:**

Notably, the biological consequences observed for MM and AM titanium were rather similar, considering the differences in used materials, production techniques, and subsequent surface morphologies. Carefully applied SAE can also optimize the surface characteristics of additive manufactured titanium for an improved implant performance, with storage conditions critically influencing surface wettability and bioactivity.

## 1 Introduction

Titanium is widely used for dental implants due to its high biocompatibility with both bone and gingival tissues, as well as its ability to undergo osseointegration. In clinical applications, both commercially pure titanium (cp-Ti) and titanium alloys such as Ti-6Al-4V are employed, with cp-Ti being the most commonly used material and widely regarded as the standard for dental implants (Nicholson J., 2020). Although cp-Ti can be processed additively, it poses certain challenges, including increased oxidation sensitivity and less favorable melt pool dynamics, which can lead to porosity and microstructural defects (Lee et al., 2022; Yamanaka et al., 2019). In contrast, the alloying elements in Ti-6Al-4V improve process stability and mechanical performance (Choi et al., 2017). Therefore, this alloy is widely regarded as the most used material for additively manufactured titanium components (Dutta and Froes, 2025). Furthermore, it is an established biomaterial that meets medical regulatory requirements and is clinically approved for both orthopedic and dental applications (Geetha et al., 2009). As a result, additively manufactured Ti-6Al-4V implants show translational potential, offering a reliable balance between processability and clinical performance (Aufa et al., 2022).

Additive manufacturing (AM) has advanced over recent years, offering opportunities for the production of complex and customized parts. Unlike conventional machining processes, AM enables the layer-by-layer fabrication of components directly from digital models (Ji et al., 2019; Wong and Hernandez, 2012). This approach is particularly advantageous in the medical field, where patient-specific implants can be designed and produced to match individual anatomical conditions (Salmi, 2021; Zadpoor, 2017). Laser Powder Bed Fusion (LPBF), a prevalent AM technique, allows for the precise manufacturing of titanium implants with complex geometries and optimized mechanical properties (Ferraris and Spriano, 2021; Haase et al., 2023; Joshua et al., 2024). Despite these advantages, AM-produced implants often exhibit high surface roughness in the as-built state, which necessitates post-processing to achieve optimal surface properties.

For dental implants to be successful and to minimize the risk of implant failure, the healing process represents a crucial stage (Esposito et al., 2013; Sakka et al., 2012). In this context, surface properties such as surface roughness, morphology, and wettability are particularly important, serving as valuable indicators for predicting implant success and osseointegration potential.

Surface roughness plays a crucial role in cellular responses and osseointegration. Implants with moderate roughness (Sa approximately 1µm–2 µm) have demonstrated improved bone response and osseointegration (Wennerberg and Albrektsson, 2009). Enhanced osteoblast adhesion, proliferation, and differentiation have also been observed on surfaces featuring controlled micro- and nanoscale roughness (Gittens et al., 2014). Surface wettability influences initial cellular interactions. Hydrophilic titanium surfaces enhance osteoblast adhesion and proliferation compared to hydrophobic surfaces, potentially promoting better osseointegration (Zhao et al., 2005). Surface wettability plays a critical role in the initial cellular response by influencing protein adsorption and subsequent cellular adhesion and differentiation (Rupp et al., 2014).

The surface properties of implants are altered during manufacturing and subsequent processing steps. To achieve desired surface characteristics that promote osseointegration, specific surface treatments are commonly applied. Surface treatments such as sandblasting and acid etching, also known as SLA (sandblasted, large grit, acid-etched) or sandblasting and acid etching (SAE) are well established and have been shown to enhance the healing process (osseointegration) of implants within the surrounding biological environment (Rupp et al., 2014; Kim et al., 2015; Sayin et al., 2021). Thus, precise control of surface roughness *via* SAE or similar treatments is critical for achieving favorable clinical outcomes (Rupp et al., 2018).

Surface properties of titanium implants can vary over time due to environmental factors and storage conditions. Storage conditions have been shown to influence surface wettability and bioactivity of titanium surfaces. Storage in ambient air reduces titanium surface wettability through adsorption of hydrocarbons, negatively impacting initial cell attachment and biological interactions (Att et al., 2009). In contrast, storage in isotonic solutions preserves the hydrophilicity of titanium surfaces, maintaining better biological activity compared to ambient air storage (Lu et al., 2012; Schwarz et al., 2008). Chemically modified hydrophilic surfaces stored appropriately have also demonstrated enhanced tissue integration (Schwarz et al., 2008).

Another major drawback of current implant designs is the significantly decreased success rate in medically compromised patients, highlighting a persistent gap in the field. Digital dentistry and AM offer promising solutions by enabling the customization of implant geometry and surface properties through appropriate surface treatments to better match individual patient needs (Tuikampee et al., 2024).

Although the effects of different sandblasting parameters and storage conditions on the surface characteristics of titanium have been investigated in other studies (Chen et al., 2022; Liu et al., 2025; Stoilov et al., 2022), these investigations were conducted on conventionally manufactured titanium samples. In contrast, AM titanium, despite its growing clinical relevance, has received comparatively limited attention in this context. Notably, systematic data on the response of AM surfaces to such treatments remain rare, and direct comparative analyses between AM and conventionally manufactured or machined manufactured (MM) titanium, processed under identical surface conditioning and storage protocols, are virtually absent from the current literature.

The aim of this study was to apply common SAE treatments on additive and machine manufactured titanium to directly compare the outcomes regarding modified surface properties. In this context also the subsequent effect of different storage times and conditions were considered to identify critical factors in material processing for an expected optimized implant osseointegration, which was assessed according to the behavior of primary human osteoblast cells *in vitro*.

## 2 Materials and methods

In this study, AM samples made of Ti-6Al-4V (grade 5 Eli) and MM samples made of cp-Ti (grade 4) were processed by sandblasting and acid etching (SAE). The surface characteristics were analyzed by determining the surface roughness using an optical profilometer (Micro-Prof 100, FRT GmbH, Bergisch-Gladbach, Germany) and recording scanning electron microscope (SEM) images (EVO MA10, Carl Zeiss, Oberkochen, Germany) after each processing step. The contact angles of the samples were measured after different storage times in ambient air and NaCl solution (0.9% concentration, B. Braun SE, Melsungen, Germany) using a contact angle measuring device (OCA 40, DataPhysics Instruments GmbH, Filderstadt, Germany).

For the biological investigations, primary human osteoblast cells were cultured on AM and MM titanium surfaces directly after SAE and considering storage in ambient air or NaCl solution for 6 weeks. The cytocompatibility of these surfaces was assessed by evaluating alterations in cell morphology, adhesion, and membrane integrity of primary human osteoblasts.

### 2.1 Sample preparation

**FIGURE 1:**
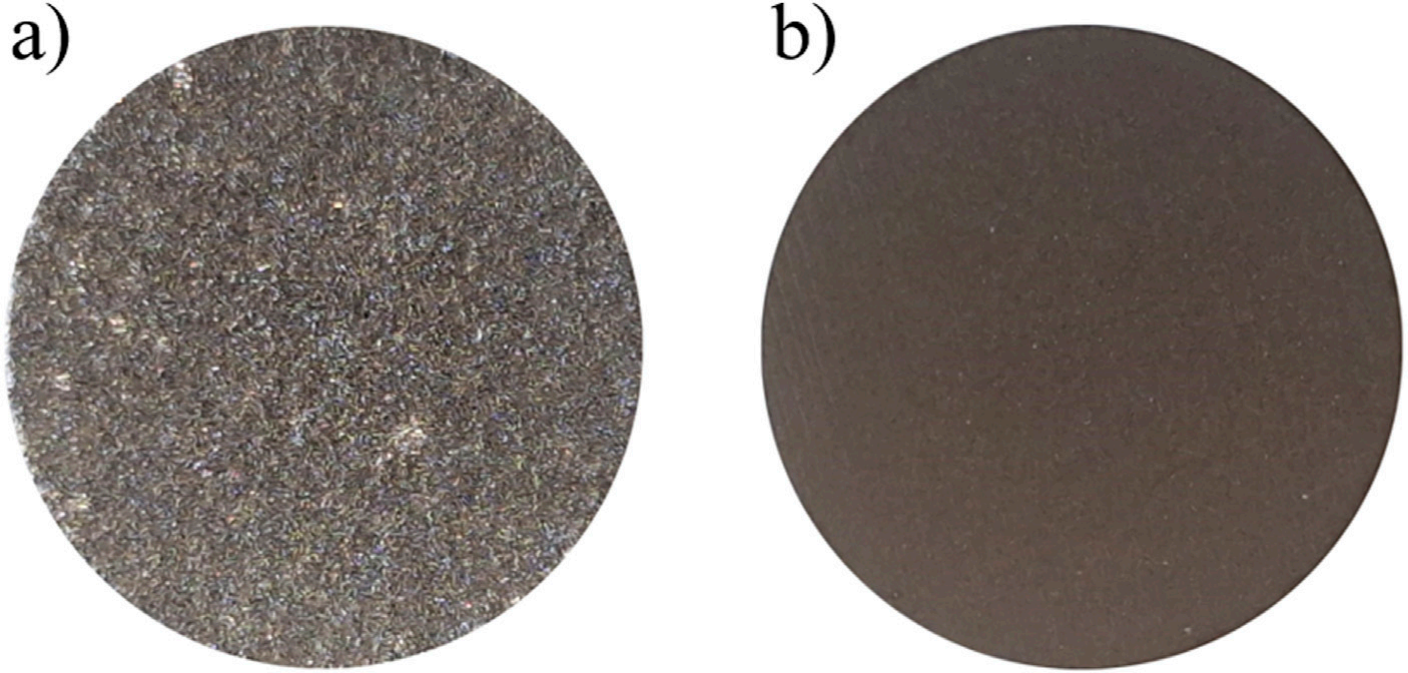
Macro image of **(a)** AM sample, **(b)** MM sample.

All samples in this study were disc-shaped and had a nominal diameter of 12 mm and a nominal thickness of 1.8 mm. The AM samples were produced using a Lasertec 12 SLM printer (DMG Mori AG, Bielefeld, Germany), which was equipped with a 400 W fiber laser (wavelength: 1,070 nm, continuous wave mode, minimum spot diameter: 35 μm). The process parameters for the contour included a laser power of 200 W and a laser speed of 1,000 mm/s, while a laser power of 175 W and a laser speed of 1,050 mm/s were utilized for the hatching. A powder (ECKART GmbH, Hartenstein, Germany) with a predominantly spherical morphology and particle sizes between 20.0 μm and 53.0 μm was used as the printing material. The samples were produced in a standing orientation, where the disk axis was aligned parallel to the building platform. To refine the microstructure and reduce residual stresses caused by the additive manufacturing process, the samples were subjected to heat treatment in accordance with ISO 20160 (2006). They were heated in an oven to 1,050 °C for 4 h and then cooled. Figure 1a shows as-built AM sample.

The MM samples made of cp-Ti with the same dimensions were produced by cutting with a linear precision saw (Brillant 220, ATM Technologies GmbH, Nienhagen, Germany) from 12 mm titanium rods (L. Klein SA, Biel, Switzerland) at 5,000 rpm and a cutting speed of 1.5 mm/min. A diamond cutting disc (Buehler GmbH, Braunschweig, Germany) with a diameter of 203 mm and a thickness of 0.9 mm was used as the cutting tool. Figure 1b shows an as-cut MM sample.

### 2.2 Sandblasting

An automated three-axis sandblasting machine from a previous work was used for sandblasting (Akbas et al., 2025; Finger et al., 2020). The machine consists of a 3D-printed frame construction kit (Reptile, Locxess-Trading, Villingen, Germany), equipped with a sandblasting unit (IP Mikro-Sandy, IP Dental Division GmbH, Haimhausen, Germany). It enables precise, controlled movement of the sandblasting nozzle in all three spatial directions using G-code programming to produce a uniformly sandblasted surface. The sandblasting paths followed a meander pattern. Further details on the setup and the influence of individual sandblasting parameters can be found in the previously publication (Akbas et al., 2025). The samples were sandblasted using corundum (Al_2_O_3_) particles with a nominal grain size of 110 μm (SHERA Werkstoff-Technologie GmbH, Lemförde, Germany). To securely hold the samples in place during the sandblasting process, double-sided adhesive tape was used to fix them to the sample holder. A forward speed of 1 mm/s, a path offset of 0.3 mm, a sandblasting distance of 15 mm, and a sandblasting angle of 90° were used for this process. Sandblasting pressures of 2 bar and 6 bar were used to generate different surface roughnesses.

After sandblasting, the samples were cleaned to remove contaminants introduced during the process. The first cleaning step involved placing the samples in an ultrasonic bath (Emmi-H30, emmi Emag AG, Mörfelden-Walldorf, Germany) with acetone (J. T. Baker, Phillipsburg, New Jersey, USA) to dissolve adhesive residues from the double-sided tape used during sandblasting. This was followed by three consecutive cleaning steps in an ultrasonic bath with distilled water, each lasting 10 min, to remove any loose sandblasting particles remaining on the surface.

### 2.3 Acid etching

The sandblasted samples were clamped upright in a Teflon holder and acid etched in a beaker with continuous stirring so that both sides of the samples were evenly exposed to the acid. A small area was shielded from the solution by clamping.

The acid solution consisted of concentrated sulphuric acid (H_2_SO_4_, 96%, 2,494,582, Fisher chemicals, Leicestershire, UK) and concentrated hydrochloric acid (HCl, 37%, 2,400,126, Fisher chemicals, Leicestershire, UK) in a ratio of 1:2. The exposure time was 10 min at a temperature of 90°C. These parameters were chosen based on preliminary investigations and literature references (Chauhan et al., 2021; Smeets et al., 2016; Wong et al., 2004). After acid treatment, the samples were immersed in ultrapure water type 1 for 5 min to neutralize acid residues and then dried under ambient conditions.

### 2.4 Storage of the samples

Following acid etching, samples were divided and stored in ambient air and NaCl solution for 6 weeks. For storage in ambient air, the samples were kept in a controlled laboratory environment. Temperature and humidity were monitored using a hygrometer at regular intervals over a period of 6 weeks, with a total of 10 measurements recorded. The recorded temperature was 24.9°C ± 0.5°C, while the relative humidity was 60.1% ± 4.2%. The samples were placed in well plates with the lid slightly open to allow air exchange with the environment. This storage was chosen to analyze the effect of typical storage in a laboratory environment which occurs between experimental steps and might lead to different results, that might get neglected in other studies.

For storage in NaCl solution, each sample was placed in an Eppendorf tube and fully immersed in the solution. The tubes were sealed and positioned upright on a stand to ensure stable conditions during storage. To prevent potential light-induced surface alterations, as described in previous studies (Belén et al., 2022; Miyauchi et al., 2002), all samples (both in ambient air and NaCl solution) were protected from light exposure by wrapping the storage containers in aluminum foil. NaCl stored samples were gently washed with distilled water without drying prior to cellular experiments to remove non-precipitated ions.

### 2.5 Experimental setup

The surface characterization of the samples was performed for every process step and storage condition using roughness measurements, scanning electron microscopy (SEM) imaging, and contact angle measurements. In addition, cytocompatibility of samples were evaluated immediately after acid etching and under storage conditions using human osteoblasts. This was estimated by evaluating the changes in cell morphology, adhesion, and membrane integrity. An overview of the different process steps, conditions, and performed investigations as well as notation of samples is presented in Table 1.

**TABLE 1:** Sample overview and notation of examined groups.

Basic material	Notation of samples			Storage conditions (6 weeks)	Assessment
	Untreated Ti samples	After sandblasting (2 or 6 bar)	After sandblasting and acid etching		
Additive manufactured titanium (Ti-6Al-4V)	AM	AM-SB2	AM-SB2-AE	Ambient air NaCl solution	Surface roughness Surface topography Surface wettability Cytocompatibility
		AM-SB6	AM-SB6-AE		
Machined manufactured titanium (cp-Ti)	MM	MM-SB2	MM-SB2-AE		
		MM-SB6	MM-SB6-AE		

### 2.6 Roughness measurement

The mean roughness value Sa was determined by using an optical profilometer (Micro-Prof 100). The measurement was performed on a measuring field of 3.2 mm × 3.2 mm (10.24 mm^2^) in the center of the sample surface, whereby 800 × 800 (640,000) data points were recorded. The software Gwyddion version 2.63 (Petr Klapetek, Brno, Czech Republic) was used to determine the average roughness. First, the raw data was adjusted using a plane correction. To minimize distortions caused by measurement artefacts, the 3σ rule was applied (Blázquez-García et al., 2021). This statistical method identifies outliers by excluding data points outside of three standard deviations (σ) around the mean. The results were determined as the mean value and standard deviation.

### 2.7 Scanning electron microscopy

After roughness measurement, the samples were analyzed using a scanning electron microscope (EVO MA10). A magnification of 1,000x was selected to image areas of 225 μm × 300 μm. This high magnification made it possible to analyze the surface topography and microstructural changes in detail. The investigations were carried out at a working distance of 10.5 mm, using an acceleration voltage of 15 kV and a spot size of 400 nm.

### 2.8 Contact angle measurement

The contact angle measurement was conducted in accordance with DIN ISO 19403-2 (DIN EN ISO, 2025). For this purpose, a dry surface must be ensured, which is why the samples stored in NaCl were first washed with ultrapure water type 1 and then dried for 3 h in ambient air prior to contact angle assessment. This drying step ensured consistent measurement conditions comparable to the initial contact angle measurement for the ambient air storage groups. A contact angle measuring device (OCA 40) with the SCA20 software was used. Ultrapure water type 1 was used as the measuring liquid, with a drop volume of 3 μL at a dosing rate of 1 μL/s. The drop was applied to the center of the sample surface. After 10 s, an image was taken to determine the contact angle. The evaluation was done by using the manual polynomial method to precisely define the drop contour and the baseline. The measurement was performed with the camera tilted by 2° and a twofold magnification. Hot pixel correction was used for image correction.

### 2.9 Human osteoblast cell cultivation

A primary human osteoblast cell line (Lonza, Basel, Switzerland) was obtained and cultured using Alpha minimum essential medium (α-MEM medium, Pan-Biotech GmbH, Aidenbach, Germany) containing 10% fetal bovine serum (Pan‐Biotech, GmbH, Aidenbach, Germany), and 1% streptomycin/penicillin (A2212, Biochrom GmbH, Berlin Germany) at 37°C in a humidified incubator with 5% CO_2_. Cells were then trypsinized with 0.25% Trypsin/0.02% EDTA upon reaching 80%–90% cell confluence and used at passages five to seven for the experiments. The cytocompatibility of AM and MM titanium samples after SAE was estimated by evaluating the alteration in cell morphology, cell adhesion, as well as membrane integrity of the cultured osteoblast. The cell seeding protocol was followed as described in a previous publication (Gaikwad et al., 2025) with 1 x 10^4^ cells per 100 μL cell medium cultured on each sample. For controls, seeding was done simultaneously on glass coverslips of similar size. Lysis in the negative control group was attained with 1% Triton X-100 (93,416, Sigma-Aldrich, Chemie GmbH, Taufkirchen, Germany) in α-MEM. NaCl stored samples were washed with sterile distilled water prior to cellular experiments to remove non-precipitated ions.

In our study, sterilization of the samples was not performed prior to cellular testing, as conventional sterilization methods such as autoclaving or ethanol treatment were avoided to preserve the surface characteristics of samples. Since one of our objective was to investigate the influence of storage on cellular behavior, maintaining the native surface characteristics was critical. To minimize the risk of contamination, all samples were stored and handled in a clean, contamination-free environment using aseptic techniques.

### 2.10 Cell membrane integrity assay

Membrane integrity of cultured cells was estimated by measuring the lactate dehydrogenase (LDH) activity within the supernatant. After 24 h incubation, 100 µL of supernatants from each sample were transferred to a 96-well plate containing 100 µL of working solution (11,644,793,001, Roche Diagnostics, Germany). This plate was incubated at room temperature in the dark for 15 min, and the reaction was stopped by adding 50 µL of 1M HCl. The absorbance was measured using a spectrophotometer plate reader (Infinite 200 Pro, Tecan Group, Männedorf, Switzerland) at 490 nm (reference 690 nm) wavelength. The absorbance of the negative control group was set to 100% (maximum LDH release), while the positive control group (spontaneous LDH release) was set to 0%. Experimental values were normalized to this reference. A threshold of 20% was established to indicate toxicity, with values exceeding 20% interpreted as indicative of compromised membrane integrity.

### 2.11 Fluorescence staining and CLSM analysis

Adhesion and morphological changes of osteoblasts on AM and MM titanium were determined by fluorescence staining using a confocal laser microscope (CLSM, Leica TCS SP8, Leica Microsystems, Mannheim, Germany). After 24 h of cell cultivation, the samples were washed thrice with phosphate-buffered saline (PBS, Biochrom GmbH, Berlin, Germany) solution to remove the unbound cells. Attached cells were fixed using 4% paraformaldehyde for 20 min at room temperature. Cell permeabilization was achieved with 0.3% Triton X-100 for 10 min at room temperature. Fixed cells on the surface were stained with fluorescent dyes at room temperature in the dark for 30 minwith 4′, 6-diamidino-2-phenylindol (DAPI) (Sigma-Aldrich Chemie GmbH, Taufkirchen, Germany) was used for staining cell nuclei and Phalloidin green (Phalloidin-iFluor Reagent, Abcam, Cambridge, UK) for actin cytoskeleton. The working staining solution was prepared using PBS in 1:10,000 (DAPI) and 1:1,000 (Phalloidin green) dilutions. The 2D images were obtained at ×40 magnification at five different predetermined locations of the sample using a CLSM. Laser lines 405 nm (emission at 350 nm–470 nm) and 488 nm (emission at 493 nm–550 nm) were used to visualize the morphology of attached cells. All obtained images were qualitatively analyzed using a digital software program (Imaris 8.4, Biteplane, Switzerland). In addition, the cell adhesion was estimated by counting the number of cell nuclei per field of view and was normalized to the positive control group.

### 2.12 Statistical analysis

GraphPad Prism (Version 10, GraphPad Software LLC, California, USA) was used for data analysis of the primary osteoblast cell behavior. The normality of the data was assessed using the Shapiro-Wilk test, and the homogeneity of variance was verified using Levene’s test. Since the data met the assumptions of normality and equal variance, a two-way analysis of variance (ANOVA) followed by Tukey’s test was used to compare the mean between the groups. For all analyses, the level of significance was set at α = 0.05. Statistical results are reported as P-values, where *P < 0.05* was considered as a statistically significant difference, and *P > 0.05* indicated no significant difference between the compared groups.

## 3 Results

### 3.1 Roughness measurements

The results of the roughness measurements after the different processing steps are shown in Table 2. The values represent the mean and standard deviation calculated from measurements of n = 6 samples for the untreated and sandblasted conditions. These were divided into n = 3 samples each after acid etching and measured after 6 weeks of storage. Notably, the roughness values for the untreated and sandblasted MM samples (MM, MM-SB2, MM-SB6) were adopted from a previous study (Akbas et al., 2025). The methodology applied to measure roughness in the current study was consistent with the previous work, ensuring direct comparability of the results.

**TABLE 2:** Surface roughness (Sa ± standard deviation) of AM and MM samples after different processing steps and after storage after 6 weeks for the acid etched samples.

Untreated		Sandblasted		Acid etched		
Notation	Roughness Sa [μm]	Notation	Roughness Sa [μm]	Notation	Roughness Sa [μm] under ambient air	Roughness Sa [μm] under NaCl solution
AM	9.64 ± 0.85	AM-SB2	5.32 ± 0.47	AM-SB2-AE	5.10 ± 0.39	5.02 ± 0.47
		AM-SB6	3.84 ± 0.21	AM-SB6-AE	3.96 ± 0.29	4.02 ± 0.22
MM	0.46 ± 0.02^a^	MM-SB2	2.17 ± 0.07^a^	MM-SB2-AE	2.05 ± 0.51	2.17 ± 0.27
		MM-SB6	2.57 ± 0.10^a^	MM-SB6-AE	2.47 ± 0.28	2.46 ± 0.18

^a^ Akbas et al. (2025).

The as-built AM samples exhibited a relatively high roughness with an average Sa value of 9.64 µm ± 0.85 µm. Sandblasting at 2 bar resulted to a reduction of 5.32 µm ± 0.47 µm, whereas treatment at 6 bar resulted in a lower roughness with a value of 3.84 µm ± 0.21 µm. Subsequent acid etchi ng and storage in ambient air resulted in a slight reduction in roughness to 5.10 µm ± 0.39 µm in the 2 bar group. The samples stored in the NaCl solution also exhibited similar values. These exhibit a value of 5.02 µm ± 0.47 µm. In the 6 bar group, a slight increase in roughness can be seen to 3.96 µm ± 0.29 µm for the samples stored in ambient air and 4.02 µm ± 0.07 µm for the samples stored in the NaCl solution. The values between storage under ambient air or NaCl solution show almost identical values, indicating no notable influence of these storage conditions on the roughness.

In contrast, the MM samples initially exhibited a much lower roughness of 0.46 µm ± 0.02 µm. Sandblasting increased the roughness, reaching 2.17 µm ± 0.07 µm for samples sandblasted with 2 bar and 2.57 µm ± 0.10 µm for those sandblasted with 6 bar. Similar to the AM samples, acid etching had only a minor effect on roughness and a negligible difference between the storage conditions. The values remained largely comparable to the sandblasted state, measuring 2.05 µm ± 0.51 µm under ambient air and 2.17 µm ± 0.27 µm in NaCl solution for the 2 bar groups. For the 6 bar group 2.47 µm ± 0.28 µm under ambient air and 2.46 µm ± 0.18 µm in NaCl solution.

The standard deviation of the roughness of the AM was 0.85 µm, which decreased to 0.47 µm and 0.21 µm after sandblasting at 2 bar and 6 bar, respectively. Acid etching further reduced the standard deviation to 0.39 or 0.47 µm, and 0.19 µm or 0.22 µm, depending to the storage condition. In contrast, the machined surfaces initially showed a highly homogeneous surface with a standard deviation of 0.02 µm. Sandblasting increased the standard deviation to 0.07 µm and 0.10 μm at 2 bar and 6 bar, respectively. Acid etching further amplified this irregularity.

Overall, the results demonstrate that sandblasting had the most pronounced effect on roughness, with higher sandblasting pressures leading to smoother surfaces in AM samples and rougher surfaces in MM samples. In contrast, acid etching had only a minor influence on surface roughness, with values remaining largely similar to those observed after sandblasting.

### 3.2 SEM imaging

**FIGURE 2:**
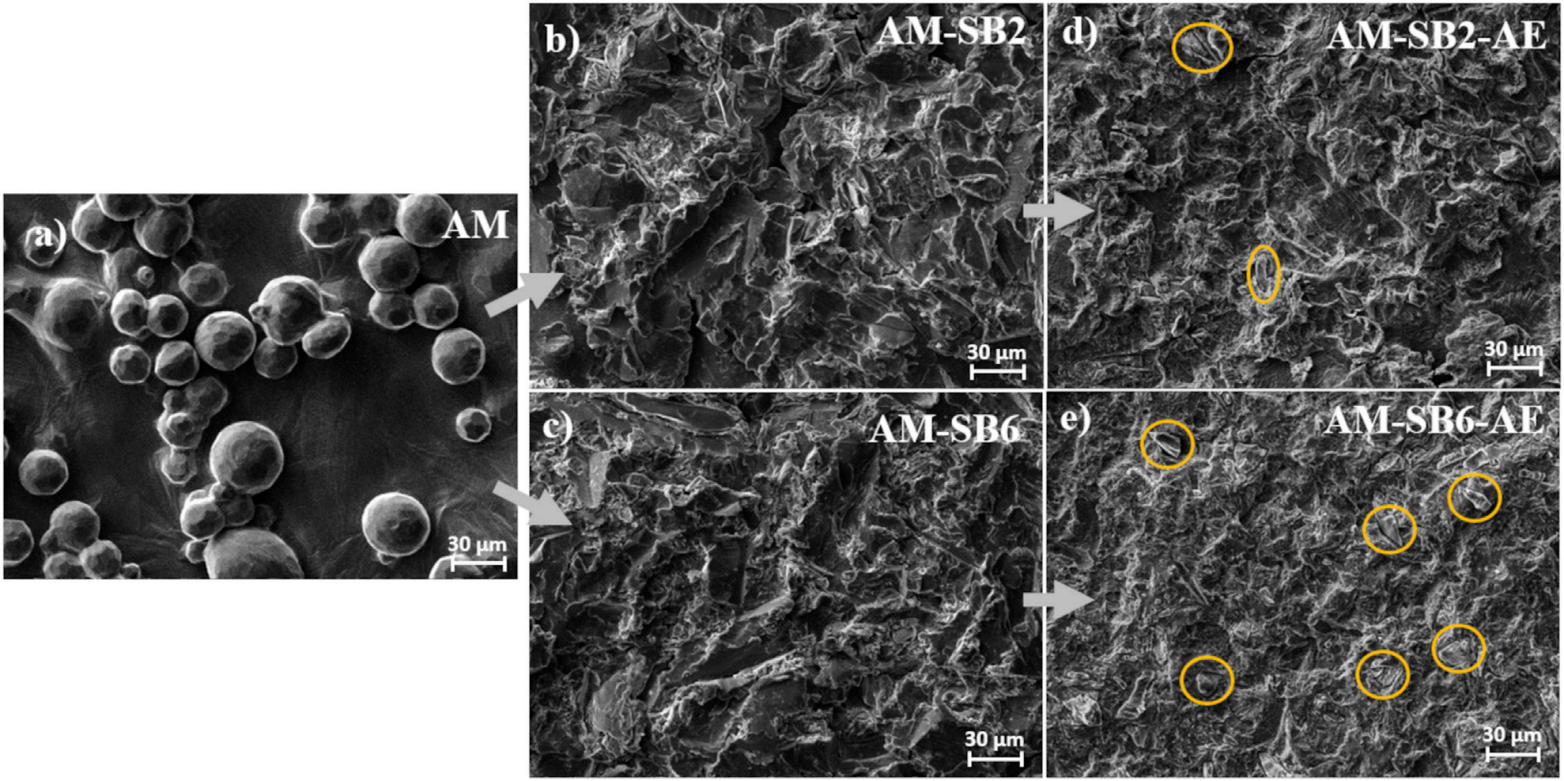
Surface morphology of AM surface **(a)** as-built state, **(b)** after sandblasting with 2 bar (SB2), with remnants of the initial spherical structures marked in red, **(c)** after sandblasting with 6 bar (SB6), **(d)** after SB2 and acid etching (AE), with embedded corundum artefacts marked in yellow and **(e)** after SB6 and AE, with embedded corundum artefacts marked in yellow.

Figure 2 illustrates the surface morphology changes observed under SEM after the different processing steps.

In the AM condition (Figure 2a), the surface exhibits a relatively smooth texture interspersed with partially melted spherical particles. These spheres originate from the printing powder that fused to the outermost surface during the LPBF process. The presence of these spherical structures is in accordance with the high roughness measured in the previous section.

Following sandblasting at 2 bar (Figure 2b, AM-SB2) a transformation of the surface morphology is evident. The originally spherical surface features are largely obliterated, replaced by an irregular pattern of ridges and grooves oriented in multiple directions. The roughness measurements align with these observations, indicating a reduction in surface irregularities.

**FIGURE 3:**
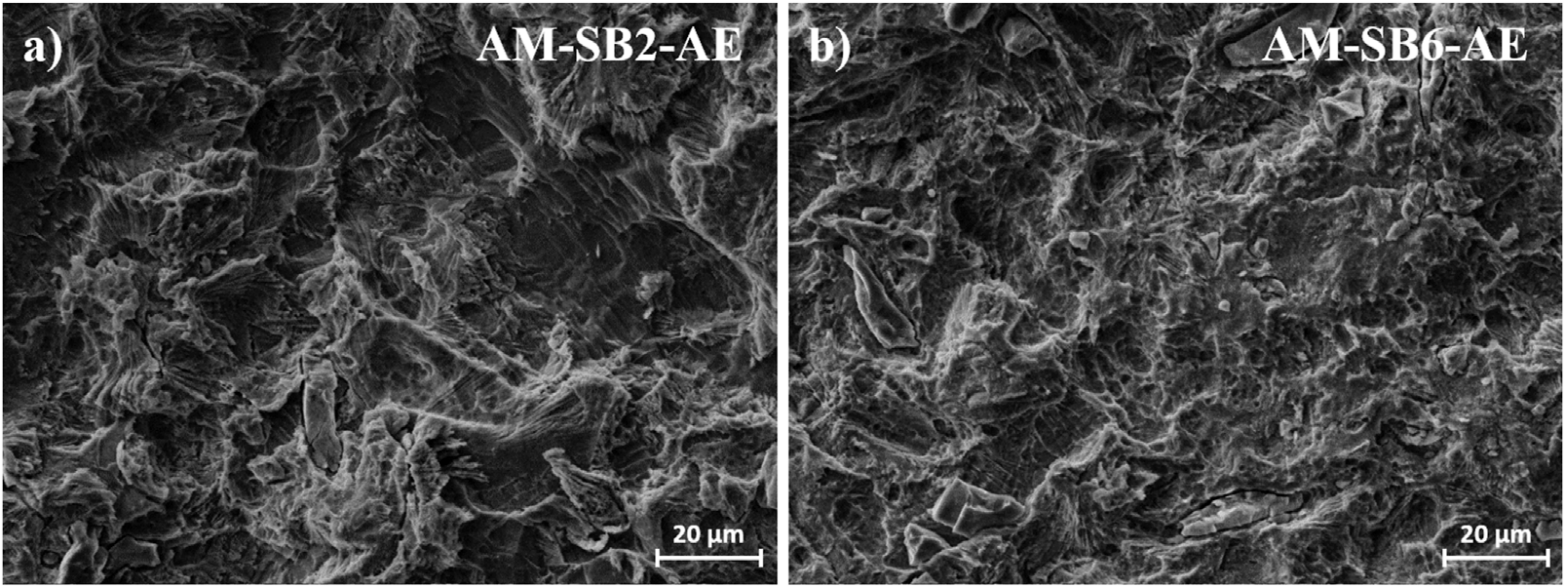
Surface morphology of AM surface after SAE for **(a)** AM-SB2-AE, **(b)** AM-SB6-AE.

After the subsequent acid etching step (Figure 2d, AM-SB2-AE), additional etching-induced irregularities become apparent, as shown in Figure 3a with higher magnification. Furthermore, embedded corundum (Al_2_O_3_) particles, introduced during the sandblasting process, become visible after etching, as marked in yellow in Figure 2d. This observation is further supported by energy-dispersive X-ray (EDX) analyses (Supplementary Figures S1 and S2), which showed noticeably stronger aluminum and oxygen signals in the areas attributed to embedded particles. Although aluminum is also a component of Ti-6Al-4V, the pronounced intensity of these peaks compared to the surrounding SAE-treated titanium surface indicates the presence of corundum residues introduced during sandblasting. These particles were not visible after sandblasting but emerged following the etching process. The roughness values remain consistent with those measured after sandblasting, indicating that acid etching had a minor influence on surface roughness.

Sandblasting at 6 bar (Figure 2c, AM-SB6) results in a surface morphology similar to that observed at 2 bar, but with more pronounced ridges and grooves. Notably, the spherical surface features that were partially retained at 2 bar are no longer visible at 6 bar, suggesting that the higher impact energy facilitated their complete removal. The increased sandblasting pressure leads to a more uniform and level surface, which is in agreement with the roughness trends described earlier.

After acid etching (Figure 2e, AM-SB6-AE), the irregular etching patterns induced by the chemical treatment are similarly observed on the surfaces subjected to 6 bar sandblasting (Figure 3b). However, a greater number of embedded corundum particles is observed compared to the surface sandblasted at 2 bar. The roughness values remain in line with those observed after sandblasting, further indicating that etching had little impact on the overall surface characteristics.

**FIGURE 4:**
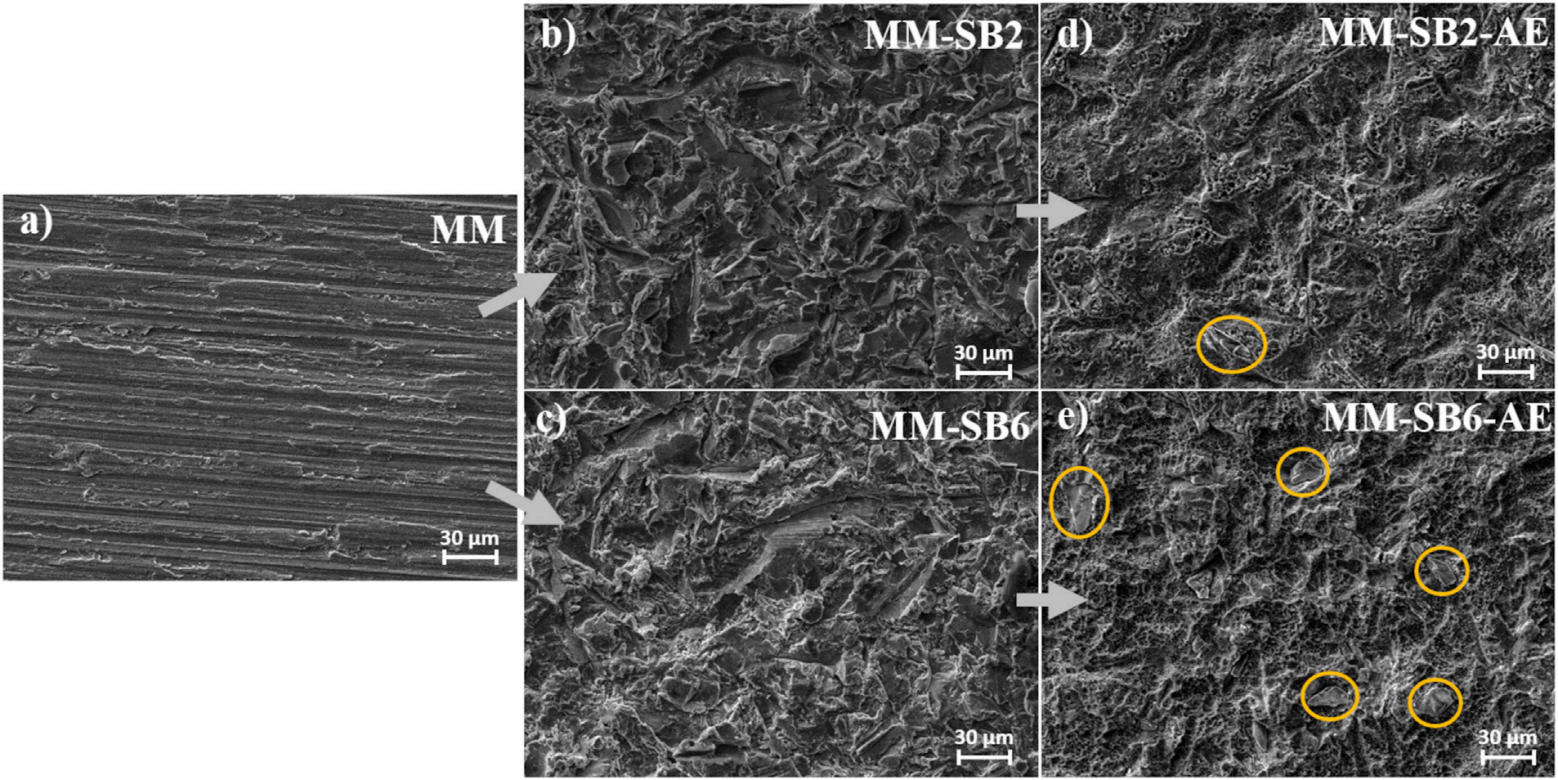
Surface morphology of the MM surface **(a)** as-machined, **(b)** after sandblasting with 2 bar (SB2), **(c)** after sandblasting with 6 bar (SB6), **(c)** after sandblasting with 2 bar and acid etching (SB2 and AE), with embedded corundum artefacts marked in yellow, and **(e)** after after sandblasting with 6 bar and acid etching (SB6 and AE), with embedded corundum artefacts marked in yellow.

The SEM images in Figure 4 depict the morphological changes in the MM samples following the different processing steps.

The MM surface (Figure 4a) exhibits a relatively smooth texture dominated by parallel lines, characteristic of the precision cutting process used to manufacture the samples. This observation is consistent with the roughness measurements, which indicate a low initial surface roughness.

Upon sandblasting at 2 bar (Figure 4b, MM-SB2), notable alterations in surface morphology are observed. Similar to the AM samples, ridges and grooves emerge in multiple orientations. The roughness analysis is in accordance with these changes, showing an increase in surface texture. Sandblasting at 6 bar (Figure 4c, MM-SB6) further enhances these topographical features, resulting in a rougher surface.

**FIGURE 5:**
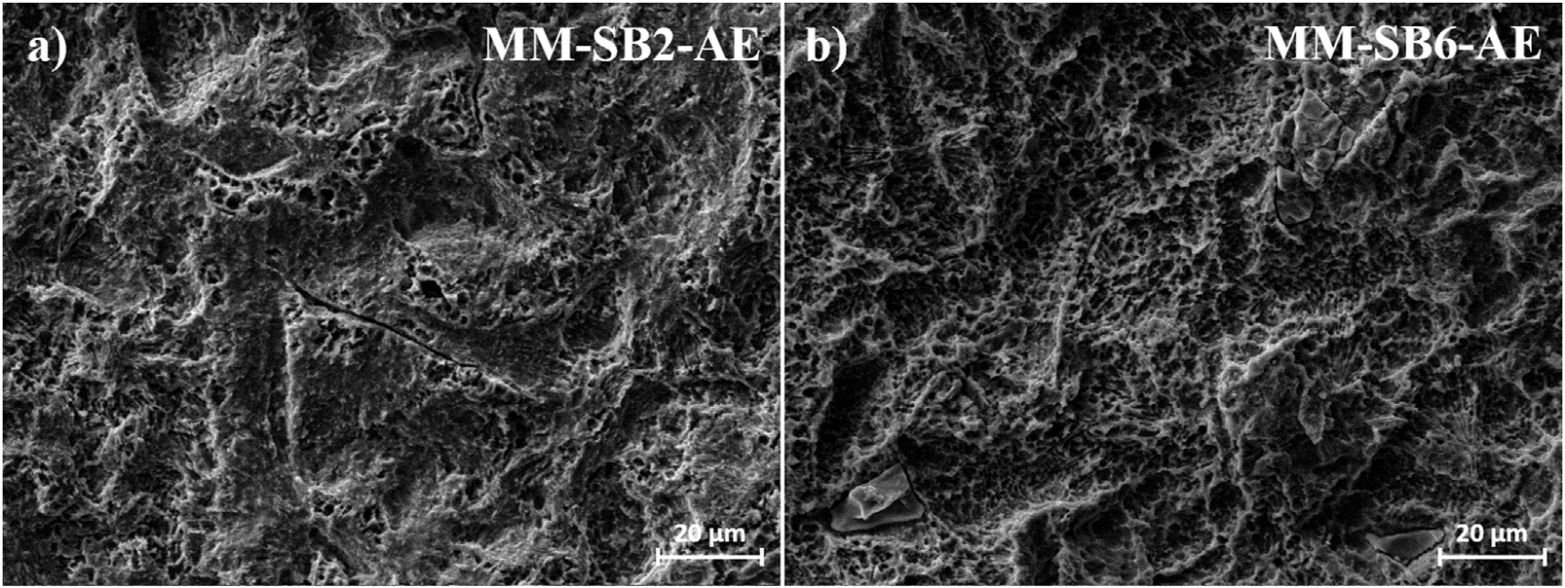
Surface morphology of AM surface after SAE for **(a)** AM-SB2-AE, **(b)** AM-SB6-AE.

Following acid etching (Figure 4d, MM-SB2-AE, and 4e, MM-SB6-AE), the primary surface structure remains largely preserved, with the addition of irregular etching patterns (Figure 5). Embedded corundum artifacts, introduced during the sandblasting process, become visible post-etching. These particles, which were not detectable after sandblasting, emerge particularly in the MM-SB6-AE samples (Figure 4e). As with the AM samples, the roughness measurements remain consistent with those recorded after sandblasting, showing only minor deviations.

### 3.3 Contact angle measurements

The results of the contact angle measurements after the different processing steps are shown in Table 3. As with the roughness data, the contact angle values for the untreated and sandblasted MM samples (MM, MM-SB2, MM-SB6) were also based on a previous study (Akbas et al., 2025). The same experimental protocol and measurement setup was used to maintain consistency and comparability with the results obtained in this study.

**TABLE 3:** Contact angle of AM and MM samples after different processing steps. The values represent the mean calculated from measurements of n = 3 samples for each condition.

Contact angle [°]							
Untreated		Sandblasted		Acid etched and stored for 6 weeks in ambient air		Acid etched and stored for 6 weeks in NaCl	
AM	91.2	AM-SB2	71.8	AM-SB2-AE	0–78.4^b^	AM-SB2-AE	0
		AM-SB6	70.0	AM-SB6-AE	0–84.1^b^	AM-SB2-AE	0
MM	91.1^a^	MM-SB2	134.5^a^	MM-SB2-AE	0–67.9^b^	AM-SB2-AE	0
		MM-SB6	135.1^a^	MM-SB6-AE	0–72.8^b^	AM-SB2-AE	0

^a^ Akbas et al. (2025).

^b^changes in contact angle shown in Figure 6 for samples stored in ambient air over a period of 6 weeks.

The contact angle measurements of the AM samples showed that the as-built surfaces exhibited a mean contact angle of 91.2°, indicating a slightly hydrophobic behavior. After sandblasting, a noticeable reduction in the contact angle was observed. Samples treated at 2 bar (AM-SB2) showed a mean contact angle of 71.8°, while those treated at 6 bar (AM-SB6) exhibited an even lower contact angle of 70.0°.

The contact angle measurements for the MM samples were obtained in a previous study (Akbas et al., 2025), also marked as ^a^ in Table 3 for clarity. The MM surfaces exhibited a mean contact angle of 91.1°, similar to the AM samples in the as-built condition. After sandblasting, an increase in the contact angle was observed. Samples treated at 2 bar (MM-SB2) showed a mean contact angle of 134.5°, while those treated at 6 bar (MM-SB6) exhibited a contact angle of 135.1°. No changes in contact angle were observed over time for the untreated and sandblasted samples.

**FIGURE 6:**
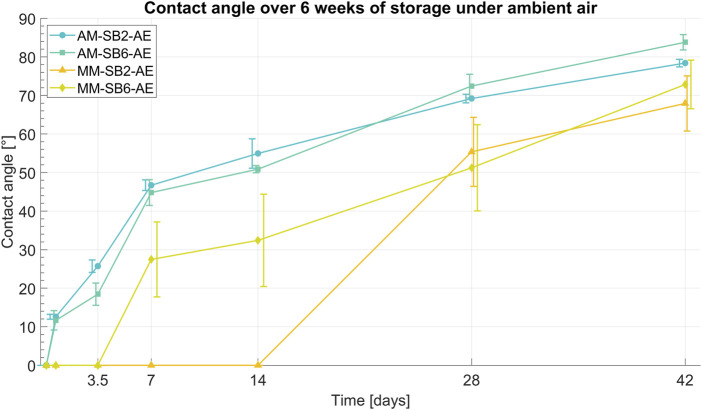
Development of the contact angle over 42 days of storage under ambient air conditions for AM-SB2-AE, AM-SB6-AE, MM-SB2-AE, and MM-SB6-AE. Representative data of mean values and error bars based on three measurements. The errors bars have been slightly shifted relative to the data points to reduce overlap, achieving a better visibility.

Following acid etching, the titanium surfaces became fully wetted, which is interpreted as a contact angle of 0°. However, during storage in ambient air, a gradual increase in contact angle was observed over a period of 6 weeks, indicating a loss of hydrophilicity. This time-dependent change in wettability is illustrated in Figure 6.

The contact angle measurements of AM-SB2-AE and AM-SB6-AE showed an initially highly hydrophilic surface 3 h after acid etching. Most samples exhibited complete wettability (contact angle of 0°), except for a single sample in of the AM-SB6-AE group, which showed a contact angle of 13.1°, resulting in a mean initial value of 6.6° for this group.

After 16 h of storage in ambient air, a noticeable increase in contact angle was observed in both groups, with mean values of 12.6° for AM-SB2-AE and 11.7° AM-SB6-AE. Over the following days, the contact angle continued to increase, initially at a more rapid rate before stabilizing with a slower progression. After 1 week, AM-SB2-AE reached an average contact angle of 46.7°, while AM-SB6-AE showed a similar trend with 44.8°. The continuous increase persisted, with both groups exhibiting a nearly identical trend in contact angle progression over the entire storage period. After 4 weeks, the contact angles reached 69.2° for AM-SB2-AE and 72.4° AM-SB6-AE, indicating a slower rate of change. At the end of the 6-week storage period (42 days), the final contact angles were measured at 78.4° for AM-SB2-AE and 83.8° AM-SB6-AE.

Similar to AM-SB2-AE and AM-SB6-AE, the contact angle measurements of MM-SB2-AE and MM-SB6-AE indicated an initially highly hydrophilic surface 3 h after acid etching, with complete wettability observed in all samples. For MM-SB-6AE, the first noticeable increase in contact angle was observed after 1 week, reaching 27.5°. The contact angle continued to increase over time, initially at a stronger rate, before slowing down in the later stages of storage. In contrast, MM-SB2-AE remained completely wettable for the first 28 days, with the first measurable increase occurring after 4 weeks, when the contact angle reached 55.4°. From this point onward, the increase in contact angle continued, following a trend similar to MM-SB6-AE. At the end of the 6 weeks storage period, the final contact angles of both groups had converged to similar values, with 67.9° for MM-SB2-AE and 72.8° for MM-SB6-AE.

Overall, all samples stored in ambient air exhibited a comparable increase in contact angle after 6 weeks of storage, with final values in a similar range. However, the contact angles of AM-SB2-AE and AM-SB6-AE remained slightly higher than those of MM-SB2-AE and MM-SB6-AE.

The samples stored in NaCl solution after acid etching also showed an initially highly hydrophilic surface, with all samples exhibiting complete wettability (contact angle = 0°). This hydrophilic surface property was preserved during the 6-week storage period in the NaCl solution, as all samples continued to exhibit complete wettability at the end of the observation period.

### 3.4 Cell morphology, adhesion, and membrane integrity

**FIGURE 7:**
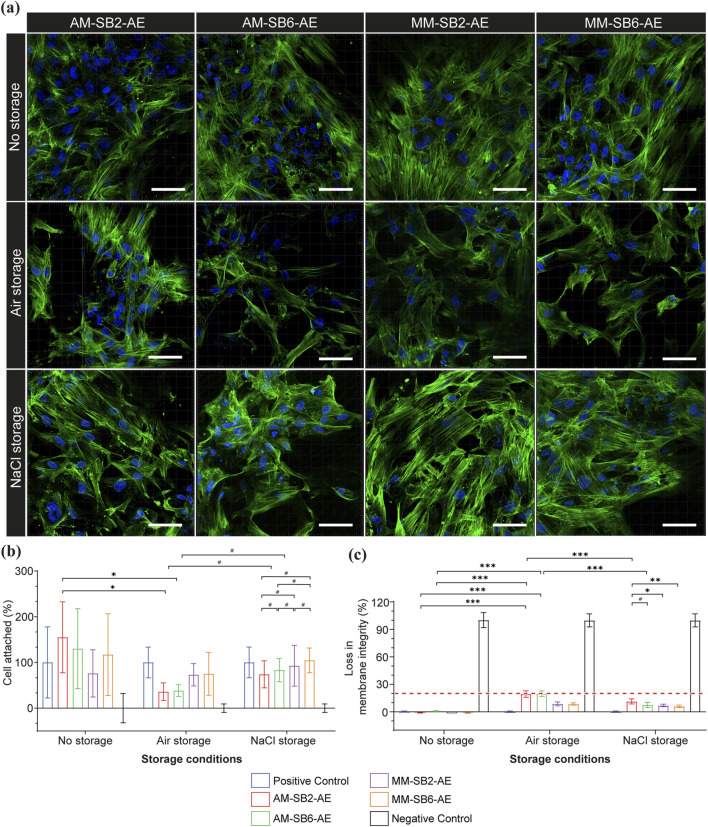
Primary osteoblast cell behavior on AM and MM titanium surfaces after SAE under different storage conditions **(a)** Representative CLSM images showing cell morphological changes in nuclei (blue) and actin (green) cytoskeleton organization. Scale bar 15 μm. **(b)** Bar graph displaying mean differences in cell adhesion. **(c)** Bar graph for loss in cell membrane integrity. Data is represented as mean ± SD. The results were statistically analyzed using two-way ANOVA with post-hoc analysis. *P* < 0.001 (***); *P* < 0.01; (**); *P* < 0.05 (*).

Figure 7 illustrates CLSM images of osteoblast cells, presenting green actin cytoskeletal filaments and blue rounded nuclei on AM and MM surfaces after SAE under different storage conditions. The titanium surfaces immediately after SAE (no storage) exhibited adequate cells with well-defined actin filaments and prominent nuclei. Moreover, the cells displayed widespread adhesion and established extensive inter-connected cytoskeletal networks that signaled a strong linkage to the underlying surface (Figure 7a). Eventually, this was consistent for both tested titanium materials with no major morphologic difference observed even after surface modification using different sandblasting pressures. The number of cell adherences on the AM titanium showed no significant difference compared to the MM titanium (*P >* 0.05), regardless of the sandblasting pressure applied in this study (Figure 7b). Moreover, these findings were supported by loss of cell membrane integrity, as the LDH activity for all tested groups remained well below the 20% threshold, indicating no cytotoxicity (*P >* 0.05), see Supplementary Table S3.

Under 6 weeks storage conditions, osteoblast cells presented prominent changes across both tested titanium in comparison to freshly etched titanium, with these effects more pronounced on AM titanium. Under ambient air conditions, the cell morphology alterations were highly noticeable across all tested groups presenting a substantial loss of cell attachment and disruption of cytoskeletal integrity. The cells showed reduced spreading and high interrupted actin organization. A similar pattern was observed in cell adhesion and membrane integrity analysis. The cell adhesion demonstrated a notable reduction compared to no storage condition, with the effects predominately influencing AM-SB2-AE and AM-SB6-AE. On other hand, the cell membrane integrity analysis for AM-SB2-AE and AM-SB6-AE revealed an increase in LDH activity further up to the threshold level. MM-SB2-AE and MM-SB6-AE also showed increased LDH activity in comparison to no storage condition, but was below 20% threshold. The *post hoc* Tukey’s test demonstrated statistically significant differences between AM and MM groups (*P <* 0.001). However, the tested sandblasting pressure (2 bar and 6 bar) did not show statistically significant differences for both AM and MM titanium (*P >* 0.05). Moreover, this increase in LDH activity for AM and MM titanium was significantly more when compared to subsequent groups under no storage condition (Supplementary Table S3).

Under NaCl storage condition the cells remained well attached but the actin filaments were marginally diffuse across tested groups with the effects further prominently characterized for AM-SB2-AE and AM-SB6-AE groups. These notable effects indicate slight loss of structural integrity and highlight the mild effects of Na^+^ and Cl^−^ ions on osteoblast morphology. Although there was a reduction in cell adhesion across all groups, it was statistically non-significant when compared to groups under no storage conditions (Figure 7b, *P >* 0.05). Furthermore, these findings were consistent with membrane integrity analysis, as LDH activity was increased but overall was below the threshold level (Figure 7c; Supplementary Table S3).

## 4 Discussion

The aim of this study was to investigate the influence of storage conditions and parameters associated with sandblasting and acid etching on the surface properties of AM and MM samples. In particular, this study aimed to determine how different surface treatments and storage conditions affect surface roughness, wettability, and subsequently osteoblast responses, contributing to an improved understanding of implant-tissue interactions.

The results revealed that the roughness of the AM surfaces in the as-built state was relatively high, while the machined surfaces exhibited lower roughness. This is consistent with previous studies that report inherent process-related roughness characteristics in AM-produced components (Greitemeier et al., 2016; Soe et al., 2024). Specifically, the study by Soe et al. found that side surfaces of additively manufactured Ti-6Al-4V samples showed roughness values of approximately 10 μm in the as-built state, aligning closely with our findings (Greitemeier et al., 2016; Soe et al., 2024). Moreover, they observed a reduction in roughness after sandblasting, which was intensified with higher sandblasting pressures. A similar trend was observed in the current study.

In contrast to previous findings, however, the present work showed slightly lower roughness values after sandblasting. This discrepancy is likely attributed to the use of larger abrasive particles in our study compared to previous work (Akbas et al., 2025).

Acid etching had a comparatively minor effect on measured roughness values, yet influenced surface topography by creating characteristic irregular etching patterns, as shown in Figures 2, 4. These etching patterns are characteristic of the SAE/SLA process and are consistently reported in the literature. For instance, Ferguson et al. described SLA-treated surfaces with crater-like micro pores and grooves, typical of the combined sandblasting with large-grit and subsequent acid etching process (Ferguson et al., 2006). Similarly, Smeets et al. highlighted the creation of micro-rough topographies with pits and protrusions that enhance cell attachment during osseointegration (Smeets et al., 2016). Furthermore, Scarano et al. demonstrated that SLA surfaces yield irregularly rounded grooves with sharp-edged micro-pores, which are advantageous for bone-implant contact during early healing stages (Scarano et al., 2017). It was also noticed, that some alumina particles become only visible after etching. This might be explained by a coverage of these particles with plastically deformed titanium, which is then removed during the etching process.

The observed decrease in standard deviation of roughness for AM surfaces after SAE suggests an increase in surface homogeneity, an effect that was particularly pronounced at higher sandblasting pressures. Conversely, the initially homogeneous machined surfaces became slightly more irregular after SAE, ultimately resembling the topography of additively manufactured surfaces. These observations indicate that the surface processing parameters influence surface uniformity, emphasizing the need for carefully controlled processing steps to achieve consistent implant properties.

The initially pronounced differences in surface roughness between the two manufacturing processes (smooth surfaces for MM samples and rough surfaces for AM samples) were noticeably reduced by the SAE treatment. While MM surfaces became rougher due to sandblasting and acid etching, the roughness of AM surfaces was noticeably reduced. As previously mentioned in the introduction, studies have shown that moderate surface roughness values (1 μm–2 µm) lead to improved osseointegration (Wennerberg and Albrektsson, 2009). Most commercially available implant surfaces are moderately rough, which emphasizes their clinical relevance (Doornewaard et al., 2016). However, there are also implants on the market that use rougher surfaces with Sa values above 2 µm (Doornewaard et al., 2016). In this study, the MM samples showed moderately to slightly rough surfaces depending on the SAE treatment. In contrast, the AM samples remained in the rough range after SAE treatment. Here, a reduction in roughness from ∼10 μm to ∼4 µm was achieved. Further adjustments of the SAE parameters such as a higher blasting pressure, the use of larger sandblasting particles or a longer blasting duration could possibly further reduce the roughness of AM surfaces and bring them closer to the clinically favorable moderate range.

For the investigations, all samples previously stored in NaCl solution were specifically washed out. This step was performed to remove possible NaCl residues that could influence the intrinsic properties of the titanium surfaces after SAE.

The contact angle measurements revealed that all samples initially exhibited highly hydrophilic surfaces immediately after SAE, as shown in Figure 6. The initial hydrophilicity can be attributed to the formation of hydrogen bonds on the titanium surface during the water storage phase following the acid etching process. As described in the study by Jiang et al. and Stepanovska et al., these hydrogen bonds form due to hydroxyl groups (-OH) created on the titanium oxide surface, which facilitate the interaction with water molecules and enhance hydrophilicity (Jiang et al., 2019; Stepanovska et al., 2020). Over time, however, a time-dependent increase in the contact angle was observed for all groups stored in ambient air, reaching values as high as 83.8° for AM-SB6-AE after 6 weeks. This transition to hydrophobicity can be explained by the gradual reaction of surface hydroxyl groups with airborne hydrocarbons, resulting in the loss of hydrogen bonds. Studies, including those by Jiang et al. and Stepanovska et al., have demonstrated that contamination of titanium surfaces by hydrocarbons from the environment is a primary cause of the loss of hydrophilicity during air storage.

In contrast, groups stored in NaCl solution maintained their hydrophilic properties. Exhibiting complete wettability after 6 weeks of storage. This preservation is likely due to the stabilization of surface hydroxyl groups in the isotonic NaCl solution, which prevents the replacement of hydrogen bonds by hydrocarbons, as supported by findings in Jiang et al. (Jiang et al., 2019).

After 6 weeks of storage in ambient air, the AM groups exhibited slightly higher contact angles than the MM groups. The initial increase in contact angle during the early storage period was more pronounced on the AM surfaces, likely due to their greater surface roughness and higher effective surface area. This increased surface area accelerates the reaction of hydroxyl groups on AM surfaces with airborne hydrocarbons, leading to a faster initial loss of hydrophilicity compared to the smoother MM surfaces. Consequently, surface roughness significantly influences wettability behavior over time. These findings are consistent with the experimental evidence presented by Stepanovska et al. (Stepanovska et al., 2020), which highlights the impact of surface morphology on wettability over time.

The cytocompatibility of AM and MM titanium was assessed exclusively for samples modified with SAE, as our surface characteristics findings demonstrated substantial improvement in surface topography, and provided an optimally rough surface conducive for osteoblast adhesion by offering increased surface area. Moreover, SAE is a widely recognized and validated surface treatment for titanium implants (Bosshardt et al., 2000; Chiang et al., 2015). Overall, the cytocompatibility findings implicated good osteoblast adhesion on both AM and MM surfaces irrespective of two different sandblasting pressures tested in the study. In agreement with previous studies (Iezzi et al., 2024), the osteoblasts showed distinctive elongated morphology and developed strong intercellular networks showing wide cell spreading. These findings are further supported by membrane integrity analysis demonstrating minimal LDH activity and further confirmed by adequate cell adhesion. This implies that underlying surface topography established with SAE facilitates optimal sites for cell attachment and provides a conducive environment for viability and growth. This is specifically relevant for AM titanium surfaces, as the SAE surface treatment removes residual powder particles, thereby reducing the negative influence on cell adhesion. Corundum particles (Al_2_O_3_) are known to contribute to third-body abrasive wear, which can lead to inflammation, pain, and ultimately aseptic loosening and implant failure (Rüger et al., 2010). Therefore, minimizing the number of residual sandblasting particles on the surface is of particular interest. As described in Section 2.2, an ultrasonic cleaning step was implemented to remove loosely attached particles from the surface. Additionally, acid etching was applied, which helped detach sandblasting particles that were weakly adhered to the titanium surface. Only sandblasting particles that had deeply penetrated the material remained on the surface after these cleaning processes. Notably, the corundum particles were not visible after sandblasting but only became apparent following the acid etching step. The results of this study indicate that the presence of these residual sandblasting artefacts did not negatively affect osteoblast growth. This observation aligns with findings from a previous study (Orsini et al., 2000) showing that titanium surface modifications with micro-roughness do not impair osteoblast activity. However, the cytocompatibility findings demonstrated optimal osteoblast behavior exclusively on freshly etched samples, without storage. The titanium samples subjected to 6-week storage conditions not only exhibited alterations in cell morphology and reduction in cell adhesion but also presented an increase in the loss of cell membrane integrity. Notably, these effects are more prominent for samples stored under amber air conditions. These findings were consistent with previously published studies (Lu et al., 2012; Hayashi et al., 2014). The negative effects on osteoblast adhesion could be attributed to time-dependent degradation of titanium surfaces, resulting in hydrocarbon or carbon contamination of the titanium surfaces when exposed to air environments (Lee and Ogawa, 2012). Hydrocarbon deposition forms thin films on the titanium surfaces, masking the inherent bioactive properties and reducing its ability to support cell adhesion and proliferation. Notably, our findings further demonstrated that these effects were more pronounced on AM titanium than on MM after SAE. This disparity may be attributed to differences in surface composition of titanium thereby making AM titanium more susceptible to hydrocarbon or carbon contamination over time. Unfortunately, presence of hydrocarbon formation cannot be validated based on present data and needs further exploration. These observations highlight the critical importance of controlling storage conditions and minimizing exposure to air to preserve the bioactivity of titanium surfaces, particularly for AM titanium implants.

In the present study, we employed a preventive strategy which presumably avoids hydrocarbon contamination (lack of hydrocarbon contamination not proven in this study) of titanium surfaces by placing them in NaCl solution for 6 weeks. Our findings demonstrated a marginal negative effect on osteoblast adhesion and morphology for these samples, compared to freshly etched surfaces, which exhibited optimal results in terms of osteoblast morphology, adhesion, and cytotoxicity. The prolonged negative effects could be attributed to changes in surface chemistry induced by prolonged storage in NaCl. This storage of titanium samples prevents hydrocarbon contamination but also results in Na^+^ and Cl^−^ ions deposition on the surface, thereby interfering with osteoblast adhesion. Tang et al. reported that titanium surfaces exposed to high concentrations of NaCl solution improve the hydrophilicity of titanium but may result in a reduction of biocompatibility (Tang et al., 2021). In contrast, few studies demonstrated that the presence of Na^+^ ions improves the biological activity of the titanium surfaces (Baszkiewicz et al., 2008; Lu et al., 2011). Nevertheless, a direct comparison of our findings with these studies is challenging, as they focused on the isolated effects of Na^+^ ions. Thus, the effects of Na^+^ and Cl^−^ ions on osteoblast growth need further exploration. Despite these effects, our findings emphasize that no-storage conditions provide the most favorable outcomes for osteoblast adhesion, and morphology, specifically for AM titanium implants. These observations underscore the importance of minimizing delays between surface treatment and implantation or to explore favorable storage conditions in order to preserve the bioactivity of titanium implants.

This study has some limitations that should be taken into account when interpreting the results. One limitation is the number of samples (n = 3 per group). Nevertheless, we did not find any major differences within the individual groups, which indicates a high reproducibility of the results. A power analysis was not conducted prior to the experiments. Although the investigated storage times of 6 weeks represent only a limited period of time, they were sufficient to see the effects of storage under ambient air and in particular to show the influence of NaCl solutions, which are frequently used in practice for storage. In this study, two sandblasting parameters (2 bar and 6 bar) were investigated with a sandblasting abrasive of a specific grain size. It is possible that other sandblasting abrasives or grit sizes could lead to surfaces with different properties, which could affect the results after etching. In addition, simple sample geometries with a flat surface (disk-shaped) were used. More complex geometries, such as those found in dental implants with a cylindrical shape and threads, were not investigated in this work. The experiments were conducted under standardized laboratory conditions, which cannot fully simulate the complex *in vivo* conditions such as mechanical loading, biological fluids and dynamic cell interactions. Future studies should take these aspects into account to further validate the results and ensure their transferability to clinical applications.

Another potential limitation of this study is the use of different titanium materials and manufacturing methods for the AM and MM groups. Specifically, the AM samples were produced from titanium alloy Ti-6Al-4V, whereas the machined samples consisted of cp-Ti. Although both materials are widely used and clinically approved for dental implants, minor differences due to material composition or manufacturing processes cannot be entirely excluded. However, literature indicates that differences in surface wettability, surface energy, and osteoblast adhesion between cp-Ti and Ti-6Al-4V, when identically treated, are minimal and generally insignificant. Studies have demonstrated comparable cellular responses and surface characteristics for both materials, suggesting that observed effects are predominantly related to surface treatments rather than the underlying material differences (Ponsonnet et al., 2003).

The results of this study provide important insights into how sandblasting pressures and subsequent acid etching influence surface properties of additively manufactured and machined titanium. Nevertheless, the effects observed in this study were based on flat-surface geometries, and further research is necessary to determine whether these findings are directly transferable to clinically relevant implant geometries with complex shapes and thread structures. Therefore, it is planned for future research work within the FOR 5250 to produce additively manufactured implants and subject them to a sandblasting and etching process in order to further optimize their surface properties. Preliminary work has been carried out on the design for additively manufactured dental implants (Kök et al., 2025). In addition, mechanical tests on additively manufactured implants were performed in preliminary studies using a chewing simulator to evaluate their long-term behavior under cyclic loading (Akbas et al., 2024). Future studies on the currently established implant surfaces would aim to evaluate the impact on cytocompatibility in more detail as well as their capacity to promote osteogenic growth and differentiation, to provide a more comprehensive understanding of their clinical potential. In addition, the 3D tissue model INTERbACT will be further developed to test sandblasted and etched additively manufactured samples in a more complex biological environment (Ingendoh‐Tsakmakidis et al., 2019). This 3D model not only provides valuable insights into the interaction of hard and soft tissue cells with the implant surface, but also facilitates a better understanding of bacterial colonization and biofilm formation on modified surfaces. It is also planned to analyze whether the investigated surfaces can be usefully combined with polyelectrolyte multilayer coatings. Initial findings on such coatings have been gained in a previous study (Andreeva et al., 2025; Andreeva et al., 2023). The aim is to achieve an improved *in vivo* reaction of dental implants through this combination and to further increase their clinical applicability.

## 5 Conclusion

• The combination of sandblasting and acid etching reduces surface roughness of rough additively manufactured samples while increasing it on smooth machined samples. Acid etching minimally affects roughness values but alters the surface microtexture.
• Surface wettability is time-dependent when stored in ambient air, with freshly processed samples showing hydrophilic behavior that transitions to hydrophobicity, particularly in additively manufactured samples. However, storage in NaCl solution preserves hydrophilicity but slightly impacts biocompatibility.
• The cytocompatibility assessment of additively manufactured and machined manufactured titanium implant surfaces modified with sandblasting and acid etching demonstrated optimal osteoblast adhesion, indicating their potential for enhanced osseointegration. However, the findings highlight the significant influence of storage conditions on osteoblast growth, particularly for additively manufactured titanium surfaces. Storage under NaCl proved to be beneficial, promoting improved cellular responses compared to direct exposure to ambient air. These results underscore the importance of carefully considering storage protocols to maximize the biological performance of titanium implant surfaces.

## Data Availability

The raw data supporting the conclusions of this article will be made available by the authors, without undue reservation.
