# Supplementary material for: Effects of sandblasting and acid etching on the surface properties of additively manufactured and machined titanium and their consequences for osteoblast adhesion under different storage conditions
Source: Front Bioeng Biotechnol. 2025 Aug 6;13:1640122. doi: 10.3389/fbioe.2025.1640122 (PMC12364894; doi:10.3389/fbioe.2025.1640122)
Supplement: Supplementary file 1 [file Supplementaryfile1.docx]

Supplementary Material

## 1 Supplementary Figures


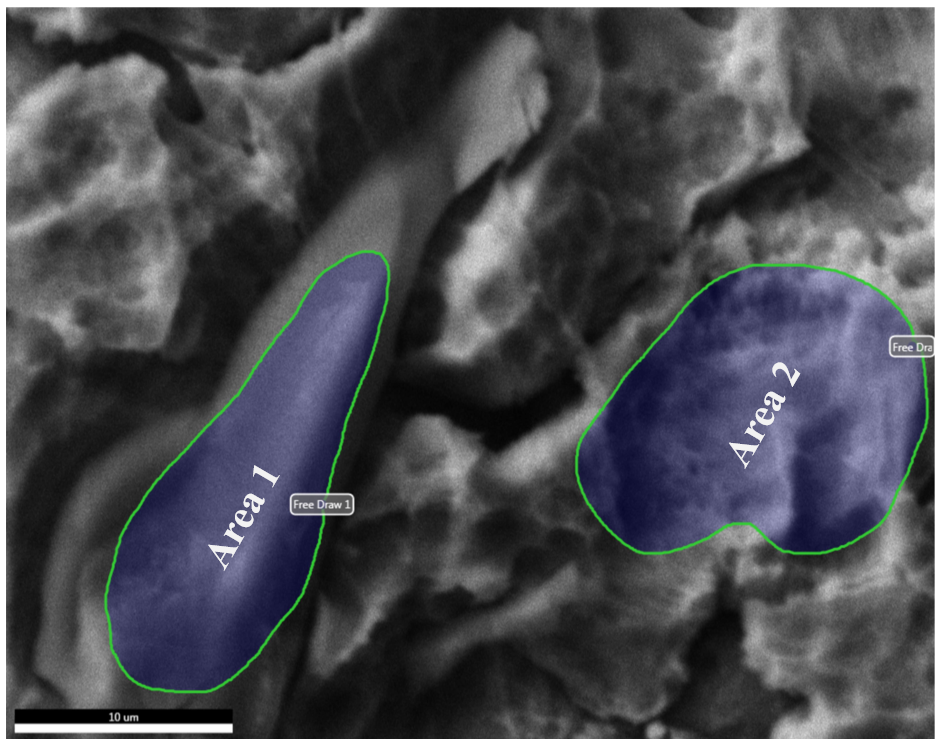


**Supplementary Figure 1:** Selected distinct regions on the AM-SB6-AE surface for energy-dispersive X-ray (EDX) analysis. Region Area 1 corresponding to the presumptive corundum (Al₂O₃), Area 1 corresponding to the presumptive SAE surface.


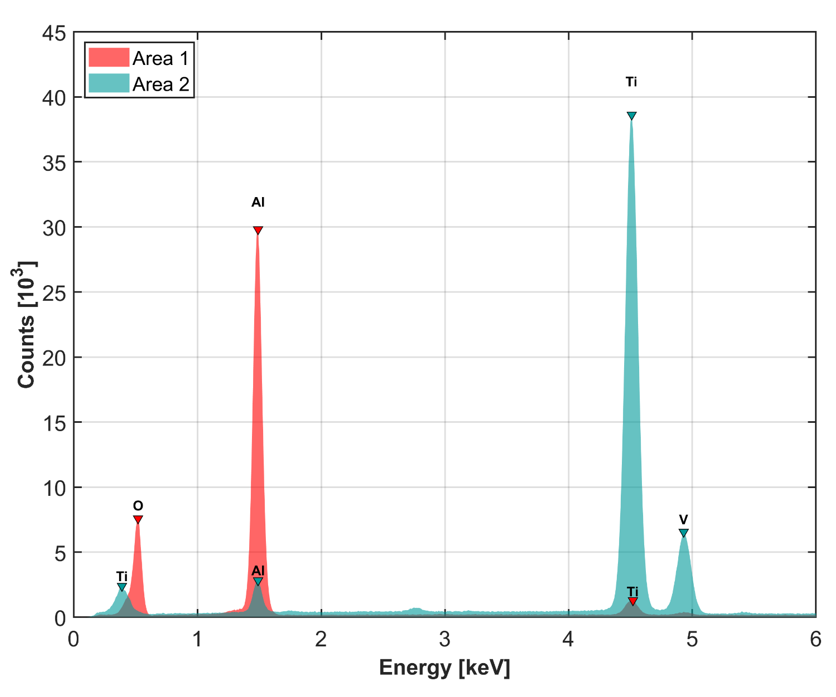


**Supplementary Figure 2:** Energy-dispersive X-ray (EDX) spectra obtained from Area 1 (presumptive corundum particle) and Area 2 (presumptive SAE surface)*.*

*
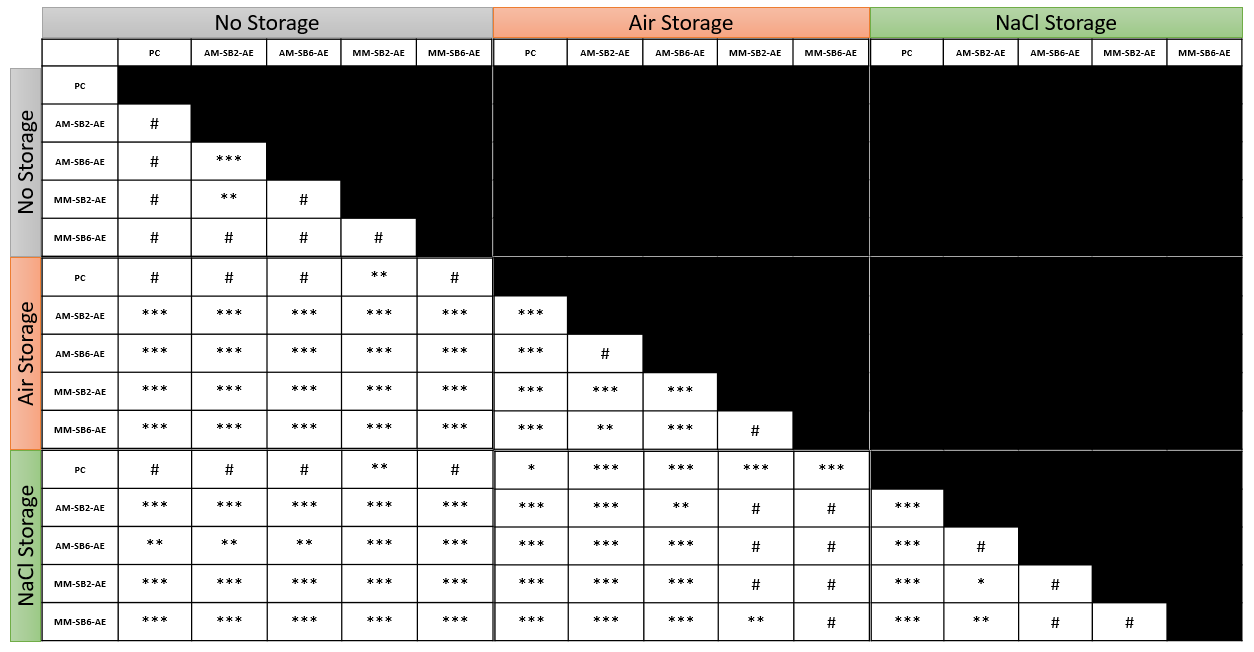
*

**Supplementary Figure 3:** Post hoc Tukey’s test showing statistical differences in loss of cell membrane integrity across tested groups under different conditions. *P* < 0.001 (***); *P* < 0.01; (**); *P* < 0.05 (*); *P* > 0.05 (#)
